# Slicing, sampling, and distance-dependent effects affect network measures in simulated cortical circuit structures

**DOI:** 10.3389/fnana.2014.00125

**Published:** 2014-11-05

**Authors:** Daniel C. Miner, Jochen Triesch

**Affiliations:** Department of Neuroscience, Frankfurt Institute for Advanced StudiesFrankfurt am Main, Germany

**Keywords:** cortical networks, graph theory, nonrandom connectivity, network topology, common neighbor, motifs, cortical slices

## Abstract

The neuroanatomical connectivity of cortical circuits is believed to follow certain rules, the exact origins of which are still poorly understood. In particular, numerous nonrandom features, such as common neighbor clustering, overrepresentation of reciprocal connectivity, and overrepresentation of certain triadic graph motifs have been experimentally observed in cortical slice data. Some of these data, particularly regarding bidirectional connectivity are seemingly contradictory, and the reasons for this are unclear. Here we present a simple static geometric network model with distance-dependent connectivity on a realistic scale that naturally gives rise to certain elements of these observed behaviors, and may provide plausible explanations for some of the conflicting findings. Specifically, investigation of the model shows that experimentally measured nonrandom effects, especially bidirectional connectivity, may depend sensitively on experimental parameters such as slice thickness and sampling area, suggesting potential explanations for the seemingly conflicting experimental results.

## 1. Introduction

Synaptic connectivity forms the anatomical substrate which gives rise to our cognitive abilities. It has been shown that much of the lateral recurrent connectivity of the cortex is significantly nonrandom. That is to say that the statistics of local connectivity do not follow that of a directed Erdős-Rényi graph, i.e., a graph in which all possible connections exist with equal and independent probability (Erdős and Rényi, 1960). For example, Holmgren et al. (2003), Song et al. (2005), and Ko et al. (2011) note the presence of greater than expected bidirectional connectivity, a feature that has been suggested as a key requirement for the sort of large-scale recurrent excitation that is seen and computation that is believed to take place in the neocortex (Douglas et al., 1995). Lefort et al. (2009), on the other hand, notes no excess of bidirectional connectivity. Song et al. (2005) additionally notes greater than expected counts of certain triangular or triadic network motifs (three-neuron connectivity patterns) (Milo et al., 2002). Yoshimura et al. (2005) examines specific microstructure, including bidirectional connections, within cortical columns. Perin et al. (2011) notes greater than expected common neighbor clustering, a phenomenon in which pairs of neurons sharing a greater number of common neighbors are more likely to be connected themselves, while Perin et al. (2013) further examines the structural implications of this above-chance common neighbor clustering. Morgan and Soltesz (2008), Litwin-Kumar and Doiron (2012), and McDonnell and Ward (2014) highlight some of the functional implications of clustering in balanced cortex-like networks. Rubinov and Sporns (2010) provides an overview of graph measures that might be applied to brain networks.The abundance of nonrandom features suggests that there may be some computational or metabolic advantage to the particular connectivity structure of the cortex. It is an open question which nonrandom features are developed as a result of direct genetic programming, neural plasticity under structured input, and spontaneous self-organization (Prill et al., 2005).

The connectome, which we take here to refer to the micro-scale, or neuron-and-synapse connectivity of the brain Sporns et al. (2005) is a detailed and difficult thing to study. Numerous methods exist for its study, including (but not limited to) increasingly detailed histological techniques (Kleinfeld et al., 2011, for example) and, more commonly, as they allow access to synaptic strengths and dynamics in addition to structure, electrophysiological recordings. We focus here on the most common implementation of the latter, involving the preparation of and recording from *in vitro* slices of cortical tissue. Though it provides more information about individual connections, the overall picture provided by electrophysiological techniques is affected by sampling biases and constraints (Seung, 2009). Traditionally, the primary concern regarding such biases and constraints has been accurate reconstruction of very small sections of circuitry. However, as techniques improve and the available sections get larger and more densely sampled, and in particular as statistical network measures are utilized more and more, it becomes important to study the effect of these biases and constraints on the network measures as well.

We examine here a simple model for horizontal connectivity in the cortex under intersomatic distance-dependent connection constraints. This simple distance-dependence results in the formation of several nonrandom features including, but not limited to, common neighbor clustering, excess reciprocal or bidirectional connectivity, and an overrepresentation of certain triadic motifs. We perform virtual slicing and sampling on this model, similar to what would be done in a physiological experiment, and examine how the results depend on slice thickness and the size of the sampling area from which cells are probed. We find, encouragingly, that such complex nonrandom features can be seeded (if not fully instantiated to the degree at which they are experimentally observed) by such simple distance-dependent phenomenon. We also find, more troublingly, that the observed representation of some of these features depends strongly on interactions of scale between the connectivity profiles, the cortical structures, and the slicing and sampling thereof. We discuss in this paper the implications of these phenomena and conclude that in order to correctly interpret data on cortical connectivity and its nonrandom features, close attention has to be paid to the exact experimental parameters such as slice thickness and sampling area.

## 2. Materials and methods

**Figure 1:**
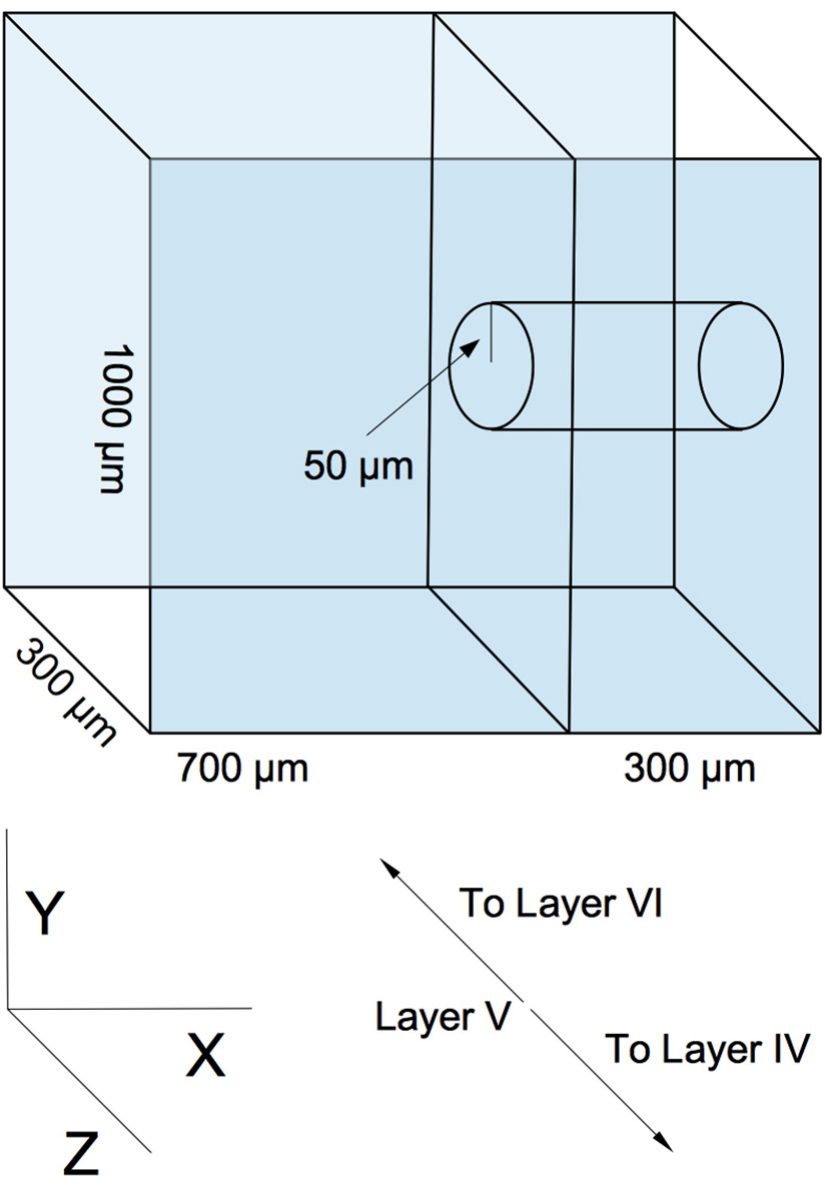
**An example of simulated slicing and sampling geometry, using a 300 μm slice and a 50 μm radius sampling area**.

Our model is designed to represent a virtual slab of cortical layer V in rodents. The slab's dimensions are 1000 × 1000 μm, with a thickness of 300 μm (the lattermost dimension describing the approximate thickness of layer V of the rodent cortical sheet (Schüz and Palm, 1989) (see Figure 1). We assume a cortical neuronal density of at least 20000 excitatory neurons per cubic mm, resulting in a total population of 6000 neurons, which are populated into the volume in a random, uniform fashion. This is a slight reduction in neuronal density from biological values, but is sufficient to demonstrate the phenomena we wish to explore and is necessary for rapid computational tractability. Though is is known that horizontal cortical axonal projections can reach lengths of several millimeters (Hirsch and Gilbert, 1991), we choose to focus on local, sub-millimeter connectivity, as this is the scale of the microstructure typically being examined in network measure studies of cortical wiring. Various connectivity models, ranging in complexity from simple piecewise dense and sparse connectivity radii (Voges et al., 2010a,b) to detailed reconstructions based on axonal and dendritic structure (Stepanyants et al., 2008; Kleinfeld et al., 2011), have been produced from experimental data. We select a continuous radial function for distance-dependent connectivity as solution between these two extremes. Our profile is a Gaussian with a half-width of 200 μm. This particular profile is chosen as a middle ground between the results of Song et al. (2005), who find no distance dependence up to a scale of 80–100 μm, and the results of Holmgren et al. (2003) and Perin et al. (2011), who find exponential distance dependence at a scale of 150–300 μm. The Gaussian compromise coarsely approximates both the flat top of the former result and the decay of the latter.

To produce the model graph, first, a 6000 × 6000 element distance matrix is constructed, with each element representing the euclidian distance between each pair of neurons. The boundary conditions are non-periodic, corresponding to slice boundary truncation. The connectivity profile function is then applied to each element, producing an unnormalized probability matrix, with each entry representing the pairwise connection probability. Self-connection probabilities are set to zero. The matrix is flattened into a vector and then the cumulative sum of the vector is taken and normalized, producing a cumulative distribution function (CDF). A look up table map is generated mapping each interval in the CDF to a particular pair of neurons.

The network is treated as a directed graph. A global connection fraction *F*_*C*_ is chosen upon model initialization, and the model is populated by generating random numbers in the interval [0,1] against the CDF and instantiating the edge mapped to the CDF interval in which each random number falls (rejecting already-instantiated edges) until the total number of edges reaches *N*_edges_ = *F*_*C*_ × (*N*^2^_nodes_ − *N*_nodes_).

Two sequential reduction procedures are then performed on the graph in order to simulate experimental sampling of the network. The first procedure simulates slicing. The virtual volume of the network is truncated along the X axis in Figure 1 to correspond to the dimensions of a typical slice (50–500 μm, depending on the experiment). Edges and nodes that fall outside the truncation region are eliminated from the graph. The second procedure roughly simulates probing and sampling. In this procedure, a subset of nodes *N*_sample_ are randomly selected from a centered cylindrical volume within the slice of radius *r*_sample_ (50–300 μm, depending on the experiment), and a subgraph is constructed from these nodes and their respective edges. This subgraph is then taken to be equivalent to an electrophysiologically obtained sample. An example geometry of this virtual slicing and sampling is shown in Figure 1).

For any selected network, be it complete, a virtual slice, or a virtual sample, we compare properties against ensembles of two types of control graphs. The first control is a comparison against a purely random graph. It is a directed Erdős-Rényi graph (Erdős and Rényi, 1960) parametrized by the same number of nodes and number of edges as the selected network.

The second control is a graph that naturally and randomly attains the amount of overrepresented bidirectional connections induced by the distance dependent connectivity, but contains no higher order effects. It is essentially a modified directed Erdős-Rényi-like graph parametrized by the number of nodes and the two independent probabilities of unidirectional connections and reciprocal or bidirectional connections. More explicitly, from the model graph, the fraction of node pairs that are unidirectionally connected and the fraction of node pairs that are bidirectionally connected is calculated. A new graph is then randomly populated with the same fractions of unidirectionally connected and bidirectionally connected edge pairs in an Erdős-Rényi-like fashion. This controls against an overrepresentation of motifs driven solely by excess bidirectional connectivity while preserving overrepresentation of motifs driven by higher order or more subtle forms of clustering.

The Python package NetworkX (Hagberg et al., 2008) and a publicly available software script that counts triadic motifs in a directed graph (Levenson and van Liere, 2011) are used to assist in the construction and analysis of graphs.

We will make comparisons between different sample and slice sizes based on overall connection fraction, bidirectional connection fraction, triadic motif count, and common neighbor clustering. We will demonstrate that sampling scale has a notable effect on how such properties are observed.

## 3. Results

We select a global target connection fraction of 0.025 for the 1000 × 1000 × 300 μm layer V slab, as this produces a local connection fraction of 0.1 for a medium-sized slice and sample, as observed in numerous layer V slice studies (Thomson and Deuchars, 1997; Thomson et al., 2002). We select three slice thicknesses (in addition to the complete network) and three sampling radii with 100 neuron subsamples (except in the case of small sections, in which case the maximum number of neurons in the section is sampled). We will examine the complete network and complete slice statistics, as well as the sample statistics for each condition, and note how they vary. Unless otherwise specified, we average over five network samples.

The global connection fraction and bidirectional connection fraction for each condition is given in Tables 1, 2. We note that in general, for a given slice size, the overall connection fraction decreases with increasing sampling radius. This is an obvious result of local clustering due to the distance-dependent connection probability. Similarly, we note that as sampling radius increases, the number of bidirectional connections over chance (as compared to an Erdős-Rényi graph) increases. This is also a result of local clustering due to the distance-dependent connection probability.

**Table 1:** **Overall connection fraction (standard error)**.

**Slice size**	**50 μm radius sample**	**150 μm radius sample**	**250 μm radius sample**	**Complete section**
Complete network	0.1343 (0.0063)	0.1066 (0.0014)	0.0749 (0.0030)	0.0250 (0.0000)
500 μm slice	0.1343 (0.0063)	0.1057 (0.0024)	0.0720 (0.0012)	0.0401 (0.0001)
300 μm slice	0.1343 (0.0063)	0.1060 (0.0021)	0.0827 (0.0034)	0.0495 (0.0001)
100 μm slice	0.1343 (0.0063)	0.1151 (0.0016)	0.0936 (0.0025)	0.0566 (0.0007)

**Table 2:** **Bidirectional connection fraction (standard error) [fraction of chance – Erdős-Rényi control]**.

**Slice size**	**50 μm radius sample**	**150 μm radius sample**	**250 μm radius sample**	**Complete section**
Complete network	0.0195 (0.0042) [1.0828]	0.0124 (0.0012) [1.0962]	0.0066 (0.0014) [1.1832]	0.0020 (0.0000) [3.1705]
500 μm slice	0.0195 (0.0042) [1.0828]	0.0126 (0.0013) [1.1228]	0.0065 (0.0006) [1.2561]	0.0034 (0.0000) [2.1140]
300 μm slice	0.0195 (0.0042) [1.0828]	0.0115 (0.0010) [1.0253]	0.0084 (0.0013) [1.2185]	0.0046 (0.0001) [1.8877]
100 μm slice	0.0195 (0.0042) [1.0828]	0.0143 (0.0014) [1.0841]	0.0101 (0.0017) [1.1517]	0.0060 (0.0001) [1.8915]

**Figure 2:**
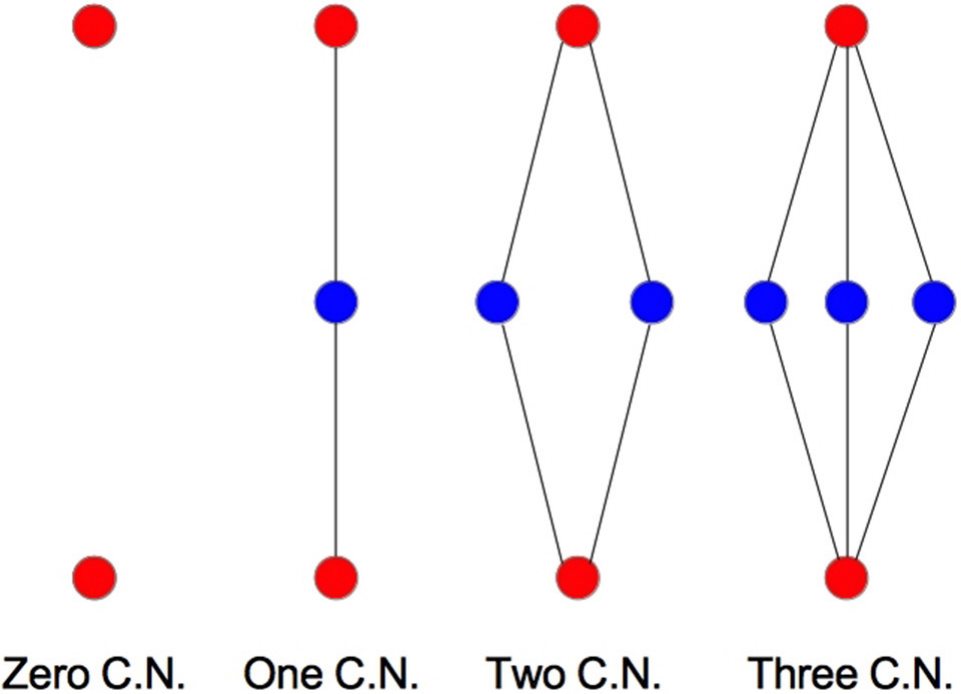
**Common neighbor clustering illustrated**. Tested nodes are red; common neighbors are blue.

**Figure 3:**
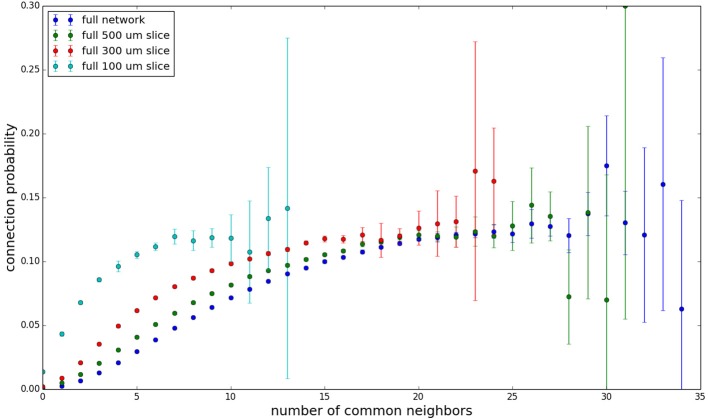
**Common neighbor clustering for complete network and slices (full sampling): pairwise connection probability as a function of number of commonly connected neighbors**. Error bars indicate standard error of the mean. Average over five populations.

**Figure 4:**
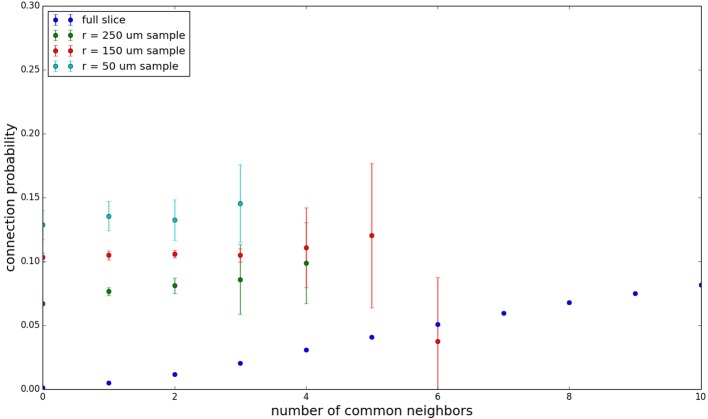
**Common neighbor clustering for 500 μm slice: pairwise connection probability as a function of number of commonly connected neighbors**. Error bars indicate standard error of the mean. Average over five populations.

**Figure 5:**
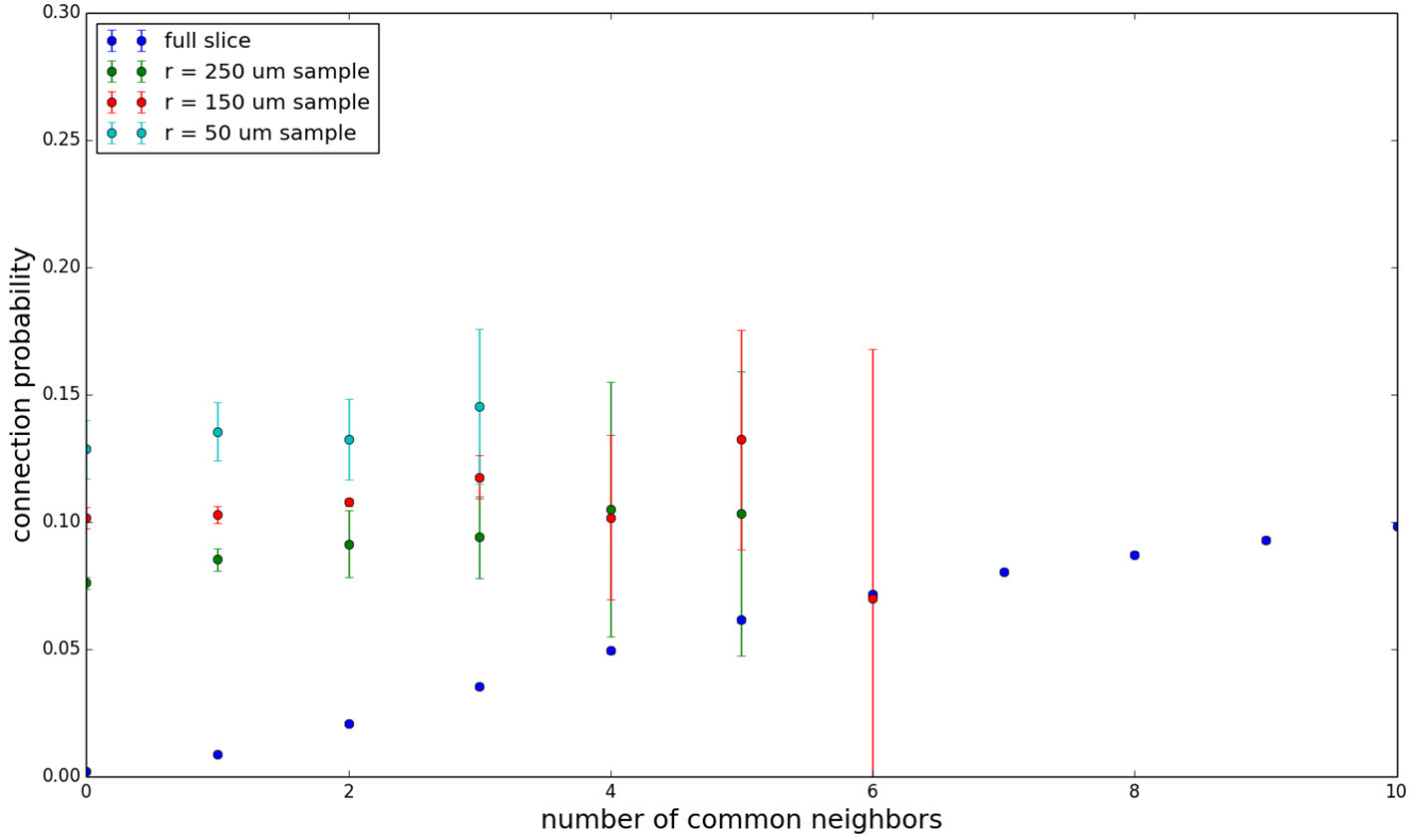
**Common neighbor clustering for 300 μm slice: pairwise connection probability as a function of number of commonly connected neighbors**. Error bars indicate standard error of the mean. Average over five populations.

**Figure 6:**
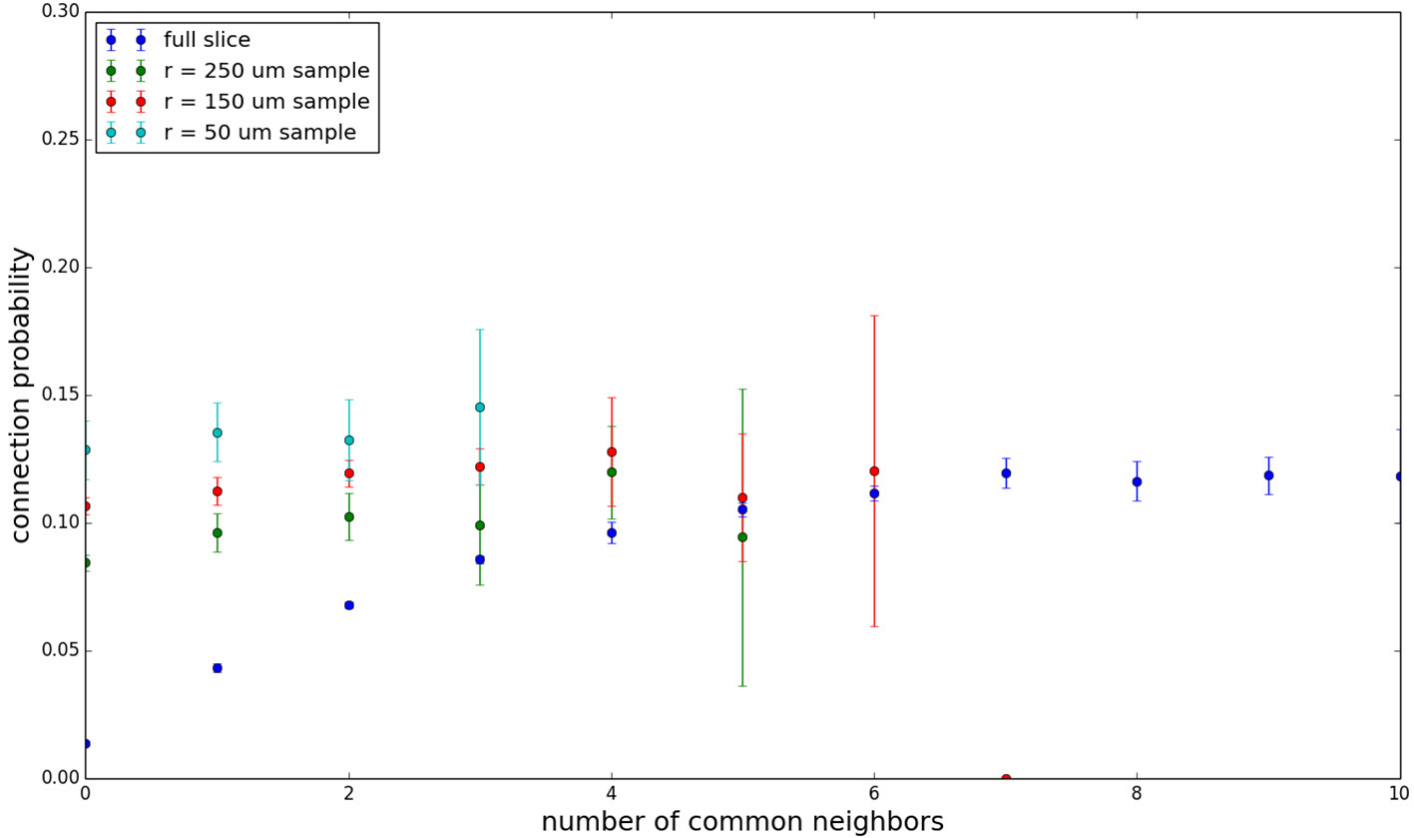
**Common neighbor clustering for 100 μm slice: pairwise connection probability as a function of number of commonly connected neighbors**. Error bars indicate standard error of the mean. Average over five populations.

We examine the common neighbor behavior in **Figures 3–6**. The common neighbor effect is measured as follows. Pairs of neurons sharing each possible number of commonly connected neighbors (up to some maximum value) are counted, ignoring directionality (see Figure 2). For each number of commonly connected neighbors, the number of connected neuron pairs is divided by the total number of neuron pairs, resulting in a connection probability conditioned on the number of common neighbors. The the steeper the slope of this measure as a function of number of common neighbors is, the stronger the effect (Perin et al., 2011). For an Erdős-Rényi graph, this common neighbor effect measure will have, on average, a slope of zero and a value equal to the overall connection probability (up until the maximum number of neighbors). Common neighbor clustering should not be confused with more traditional clustering measures (Watts and Strogatz, 1998; Fagiolo, 2007). Common neighbor effect is taken here as an undirected measure for two reasons: alignment with the convention of Perin et al. (2011), and because our simple structural model has no directional preference, and can thus make no prediction about it. In an actual biological or more complex simulated system, it is likely that in and out (to and from) common neighbor effects would produce different results, as is suggested in the supplementary material of Perin et al. (2011).

Figure 3 shows the total common neighbor effect for each entire slice. We note, firstly, that the slope of the common neighbor clustering increases with decreasing section size, and secondly, that the saturation point decreases with decreasing section size. We speculate that this occurs due to the truncation of connections that occurs upon slicing, and the resulting tendency of only nearby neurons to be well-connected. Similarly, for each individual slice thickness (Figures 4–6), the saturation point increases with decreasing sampling radius. The overall effect also becomes less pronounced for the smaller (in this case, 100 neuron) samples, as would be expected. The strength of common neighbor clustering is sensitive to both the neuronal and connection densities, and the size of the distance-dependent connection probability, particularly as it relates to the sampling scale. It is the sensitivity to the relationship between these scales that we wish to emphasize in these results.

Experimental data (Perin et al., 2011) shows an above-chance common neighbor effect stronger than the one demonstrated by our model for similar sampling conditions, suggesting the presence of additional clustering mechanisms in the cortex beyond the simple geometric ones examined in our model. One prediction our model makes is that after a linear or near-linear rise in connection probability as function of common neighbor count, the connection probability saturates for some large number of common neighbors. It can be extrapolated, despite the increased common neighbor effect seen in physiological data, that this sort of turnover and saturation effect will still necessarily occur for a large number of common neighbors given a sufficiently thorough sampling of a section of cortical tissue.

**Figure 7:**
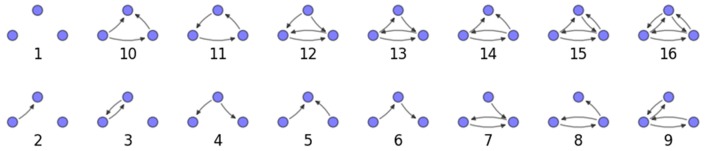
**Triadic motif key**.

**Figure 8:**
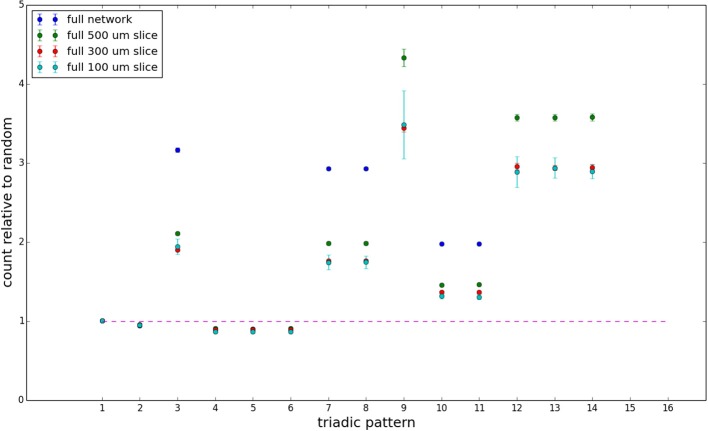
**Triadic motif counts for complete sections (full sampling)**. Error bars indicate standard error of the mean. Average over five populations.

**Figure 9:**
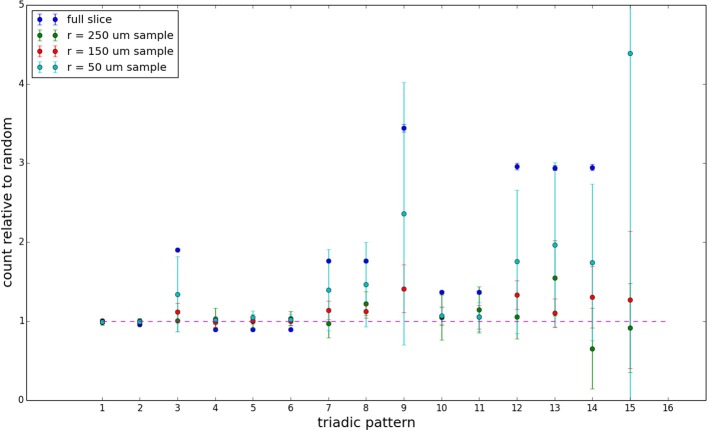
**Triadic motif counts for 300 μm slice**. Error bars indicate standard error of the mean. Average over five populations.

We examine the counts of occurrences of directed triadic motifs (possible directed triangular subgraph configurations; see Figure 7) in the simulated tissue sections compared with Erdős-Rényi random graphs for complete sections and for a sampled 300 μm slice in Figures 8, 9 (which is representative of sliced and sampled behavior, as it is observed that sliced and sampled behavior does not vary much between slice sizes; only sample radii). We note an excess of motifs with bidirectional connections. This is trivially expected from distant-dependent connection probabilities; since each direction in an edge is treated independently it will of course be the case that many minimally separated nodes will be bidirectionally connected, and, more generally that inter-group connectivity will be increased among tight groups of neurons. Furthermore, it is trivially the case that given an excess of bidirectional connections, triads containing them will be overrepresented. We wish to correct for this second effect, and do so via the bidirectionality corrected control described in the Materials and Methods section and elucidated below.

**Figure 10:**
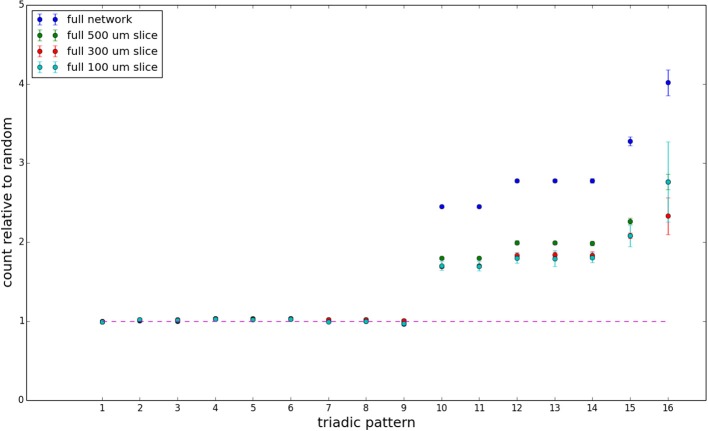
**Bidirectionally corrected triadic motif counts for complete sections (full sampling)**. Error bars indicate standard error of the mean. Average over five populations.

**Figure 11:**
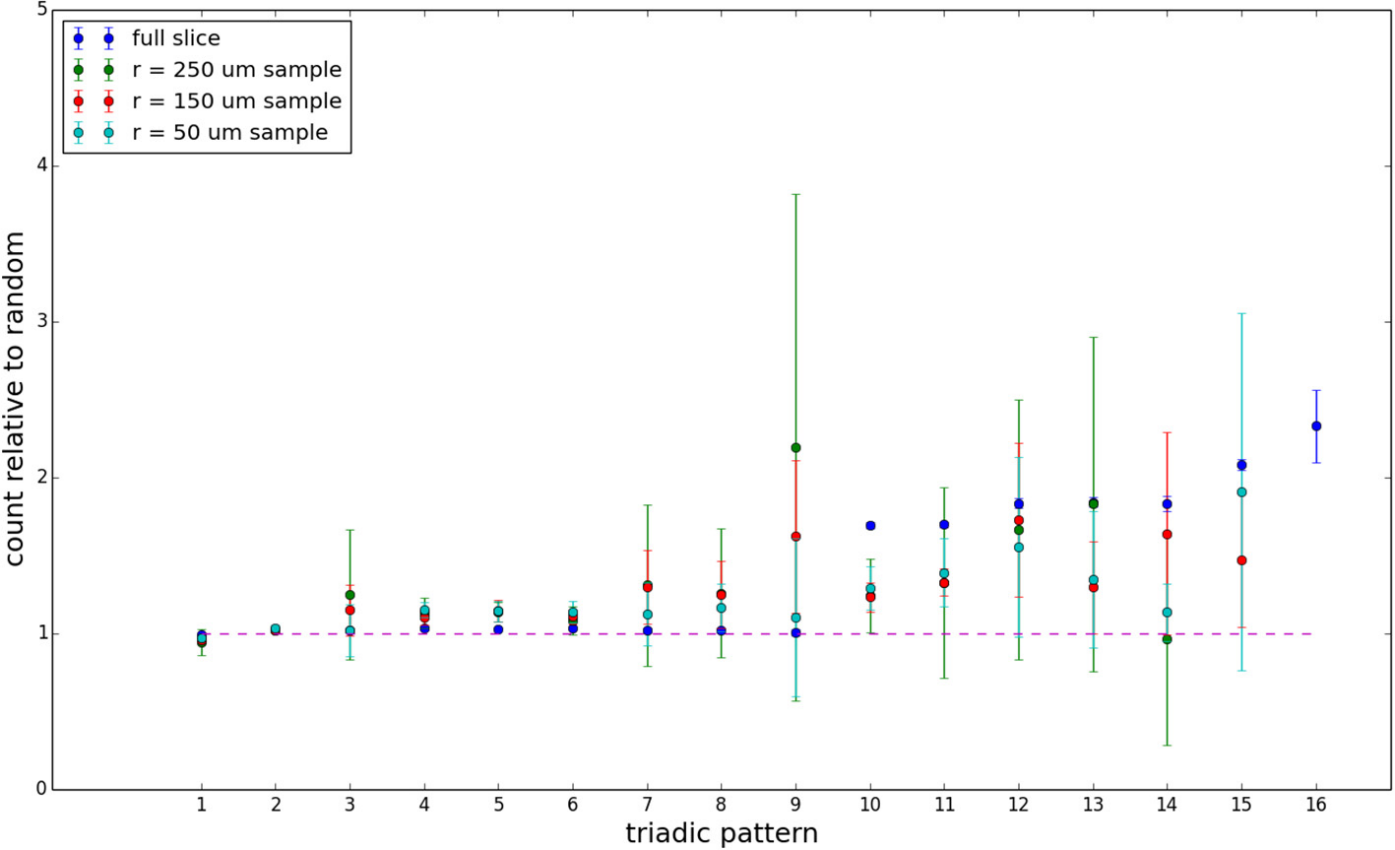
**Bidirectionally corrected triadic motif counts for 300 μm slice**. Error bars indicate standard error of the mean. Average over five populations.

We examine triadic motif counts against bidirectionality-corrected random graphs for complete sections and for a sampled 300 μm slice in Figures 10, 11. Again, sliced and sampled behavior does not vary much between slice sizes; only sample radii. We note that even after bidirectionality correction, excesses of closed-loop (i.e., connected on all sides) triadic motifs containing bidirectionally connected pairs remain. Of interest as well is the excess of closed but non-bidirectional triadic motifs (numbers 10 and 11) remaining. We note, in general, that motifs 10 -16 remain overrepresented, a phenomenon seen as well in Song et al. (2005). An underrepresentation of motif 8, which is observed in Song et al. (2005) with a similar strength to the aforementioned overrepresentations, is not seen in our model. However, the purpose of this paper is not to fully analyze the more subtle effects of distant-dependent clustering, but rather to examine the implications of similar clustering occurring at the same spatial scale as variations in sampling. We note, firstly, that as slice size decreases, the statistics of the complete slice approach the statistics of the sample. This follows logically from the fact that the sample occupies an increasing fraction of the slice by volume for a smaller slice. Along similar lines, we note that thinner slices exhibit less variation in the counts between sampling radii. For a sufficiently thin slice, one could hypothetically move from a three-dimensional to a two-dimensional reference model, approximating a sheet. We also note that post-bidirectionality correction in the control, the variation between slice sizes and sample radii is smaller than it was pre-bidirectionality correction in the control. This is a strong indicator that any motif surveys undertaken would benefit from using a bidirectionality or similar (as in Song et al., 2005) correction on the control in order to maximize consistency and universality in results.

## 4. Discussion

As we are able to access larger and denser subsamples of the connectome, complex network measures (Rubinov and Sporns, 2010) are becoming an increasingly important way of understanding both the structure and function. Such measures have already been applied to the complete connectome of *C. elegans* (Varshney et al., 2011). While elements of this study are highly telling, they do not provide a direct comparison to cortical slice studies, which are subsampled portions of a very different structure, even if the individual elements are similar. Currently, cortical slice studies provide some of the best information we have about the wiring structure of the cortex on a microscopic scale.

In order to understand this microstructure, it is very important to study and examine the statistics of connectivity at scales of tens to hundreds of μm—this will be vital to understanding the self-organizational and computational principles underlying the structure of the brain (Prill et al., 2005; Sporns et al., 2005; Seung, 2009). However, at the same time, extreme care must be taken, as relatively small variations in section size and sampling density can lead to significantly differing results, as this is also the scale at which naturally occurring simple clustering may occur, and at which the statistical transition from microstructure to macrostructure may take place as well.

It is thus of great importance that experimenters take this into account and, accordingly, provide all available information regarding neuron type and approximate density, sampling space distribution, slice thickness, and other parameters that might lead to sampling biases. Various studies of such microstructure have shown conflicting results. Reiterating, Song et al. (2005) and Holmgren et al. (2003) noted an excess of bidirectional connectivity in layer V and layer II / III, respectively. However, Lefort et al. (2009) noted no such excess. It is possible that this could be a result of sampling from different parts of the cortex which exhibit significantly different micro-organization, or that small differences in sectioning size and sampling procedure could lead to such differences. It is this latter concern that we would like to emphasize.

We have not reproduced the sampling procedures used in these studies exactly, but rather provided a generic sampling simulation from which we can gain some qualitative insight into real-world experimental results. Examining the aforementioned studies, we note that Song et al. (2005) used a 300 μm slice (Sjöström et al., 2001) with a roughly ellipsoid sampling area with radii of approximately 100 and 50 μm on the major and minor axes, respectively. Holmgren et al. (2003) also used a 300 μm slice, recording in an irregular shape out to a maximal radius of nearly 300 μm. Our model does not reproduce the high degree of excess bidirectional connections observed under these parameters, but it does result in an above-chance representation. Lefort et al. (2009), who noted no excess of bidirectional connections, used a 300 μm slice as well, further subdividing these into 100 μm sections, which would correspond to a centered recording radius of 50 μm—a radius at which our model does not exhibit a noteworthy excess of bidirectional connectivity, and suggesting an explanation for why their results appear potentially at odds with other cortical slice studies.

Our model demonstrating this concern is a simple graph model that, while it does not completely reproduce the nonrandom features noted in electrophysiological surveys, does reproduce some of them at a presumably natural scale. It is our belief that such a model provides a more reasonable, realistic, and general baseline for measuring the statistics of nonrandom cortical connectivity than a simple Erdős-Rényi graph. Certain observed complex features have been necessarily excluded to avoid an overly *ad-hoc* model. For example, our model does not reproduce the common neighbor clustering asymmetry in the in- and out-degree noted in the supplementary materials of Perin et al. (2011).

That the examined features depend so sensitively on section size in the presence of order 100 μm scale clustering should be both enlightening and concerning, particularly when most sampling procedures operate around this scale. Other factors such as neuronal type and local density almost certainly play into such effects as well. The model is not exhaustive, and numerous parameters, including the exact size and form of the connection probability profile and neuronal connection densities, could be varied. The thrust of the example provided in this paper is not to provide an exhaustive catalog of scenarios, but to demonstrate how sensitive the observed nonrandom effects of clustering mechanisms are to small variations in sampling. With this brief and simple demonstration in mind, the authors encourage experimenters to include all available information about neuronal and connection density and scale, as well as the full extent of exact sampling techniques in any study of such nonrandom features so that they can be best understood in the context of a complete graph.

## Author contributions

Dr. Miner performed the programming, analysis, and initial writing. Research direction was shared. Significant background expertise and guidance was provided by Dr. Triesch, as was significant input into the writing and revision process.

## Funding

This work was supported by the Quandt Foundation and the LOEWE-Program Neuronal Coordination Research Focus Frankfurt (NeFF).

### Conflict of interest statement

The authors declare that the research was conducted in the absence of any commercial or financial relationships that could be construed as a potential conflict of interest.

## Supplemental data

Elements of analysis source code to be released on the web via corresponding author's webpage. http://fias.uni-frankfurt.de/neuro/triesch/members/
